# Contradictions of a Vaccinationist

**Published:** 1897-09

**Authors:** E. C. Townsend

**Affiliations:** 19 Broadway, New York


### CONTRADICTIONS OF A VACCINATIONIST.

19 Broadway,

NEW YORK, September 8th, 1897.

TO THE EDITOR OF THE HOMŒOPATHIC PHYSICIAN.

SIR:—There is a precious piece of contradiction of Dr. Sweeting, *by* Dr. Sweeting, in that gentleman's evidence before the Royal British Commission, as given in THE HOMŒOPATHIC PHYSICIAN for August (pp. 299, 300), to which the learned editor of the abstract has hardly done justice, even in his note on p. 300.

At Q. 3,751 Dr. Sweeting says that he agrees with Dr. Gayton (another official advocate of vaccination) "that the vaccinated are drawn from a more neglected class," but "not that that would raise their mortality from small-pox," but at Q. 3,757 he says that "as regards progress and course of disease" (including, of course, its *fatality*) "there might be a difference according to the state of life and previous history of the patient; antecedent circumstances might have some effect upon the severity of the disease" (*i. e.*, upon its fatality), "but, in *my opinion*, would not have any effect upon the liability to attack." So that at (say) one o'clock Dr. Sweeting is of opinion that such things as dirt and malnutrition would affect the attacks, but not the fatality of small-pox; and at (say) five minutes past one the same Dr. Sweeting is of opinion that these causes might affect the fatality but not the attacks.

So that what Mr. Milne called "the unanimity of the unanimous" is here observable not only with regard to one another, but each with himself.

When will the profession of medicine repudiate these quacks, and the public open its eyes to their ignorance and cruel disregard of human suffering?

Respectfully,

E. C. TOWNSEND.

